# Antifungal and biodegradable nanoencapsulation of *Smallanthus Sonchifolius* essential oil for improved stability and sustained release

**DOI:** 10.3389/fmicb.2025.1626646

**Published:** 2025-07-01

**Authors:** Jiajie Wang, Fei Wang, Yu Zhang, Xun Li

**Affiliations:** Co-Innovation Center for Efficient Processing and Utilization of Forest Resources, National Key Laboratory for the Development and Utilization of Forest Food Resources, College of Chemical Engineering, Nanjing Forestry University, Nanjing, China

**Keywords:** poly-ε-caprolactone, *Smallanthus sonchifolius* essential oil, antifungal activity, phytopathogenic fungi, natural fungicide alternatives

## Abstract

**Introduction:**

Phytopathogenic fungi pose a serious threat to global crop productivity, necessitating sustainable alternatives to chemical fungicides.

**Methods:**

This study developed poly-ε-caprolactone (PCL) nanospheres encapsulating essential oil extracted from *Smallanthus sonchifolius* tubers (*Ss*EO) to enhance antifungal stability and efficacy.

**Results:**

The nanospheres exhibited a high encapsulation efficiency of 98%, significantly improved the photostability of *Ss*EO by protecting it from UV-induced degradation, and provided a sustained-release profile that extended its functional longevity. *In vitro* assays showed that the nanoencapsulated *Ss*EO inhibited *Sclerotinia sclerotiorum* and *Valsa mali* by 93.6% and 56.9%, respectively, while *in vivo* inhibition rates reached 94.45% and 92.5%.

**Conclusion:**

These findings demonstrate that biodegradable, photoprotective PCL nanospheres offer a promising eco-friendly strategy for plant pathogen management, reducing reliance on chemical fungicides, and minimizing environmental impact.environmental impact.

## 1 Introduction

Plant pathogens, particularly pathogenic fungi, significantly threaten global crop productivity, leading to significant reductions in both crop yields and quality each year (Fisher et al., 2012; Cheung et al., 2020). To address growing demands of the global population, effective strategies for managing a wide range of crop diseases are essential. While chemical fungicides have traditionally been the primary approach for plant disease control, their excessive use has resulted in environmental pollution, health risks, and the development of pathogen resistance, ultimately reducing their long-term efficacy in agricultural systems (Odukkathil and Vasudevan, 2013; Wang et al., 2021). In response to these challenges, researchers are increasingly focusing on plant-derived alternatives as eco-friendly solutions for crop diseases management (Wu et al., 2022). Among these alternatives, plant extracts and secondary metabolites with natural antifungal properties are being investigated as potential sources for new fungicides (Li et al., 2022; Prem et al., 2024). Essential oils, which are rich in terpenes, phenolic compounds, and aliphatic components, have demonstrated significant antibacterial and antifungal activities (Van De Vel et al., 2019).

Essential oils derived from *Asteraceae* family have demonstrated notable antifungal properties. For instance, the essential oils of *Baccharis dracunculifolia* and *Tanacetum parthenium* essential oils have been widely recognized for their ability to inhibit the growth of various plant pathogenic fungi (Lawson et al., 2020; Trindade et al., 2023). Specifically, the essential oil from *T. parthenium* contains oxygenated constituents such as camphor, farnesene, and camphene, which exhibit potent antifungal activity against a range of fungal pathogens, including *Aspergillus niger*, *Candida albicans* and *Saccharomyces cerevisiae.* Similarly, the essential oil extracted from the tubers of *Smallanthus sonchifolius* (*Ss*EO) is rich in monoterpenes and diterpenes, accounting for over 80% of its total composition, with the majority of these compounds demonstrating significant antifungal activity (Miyazawa and Tamura, 2008).

However, the practical application of essential oils is constrained by their high volatility, relatively low yields, and susceptibility to biodegradation when exposed to light, oxygen, and moderate temperatures (Meng et al., 2022). To overcome these limitations, nanoencapsulation has emerged as a promising strategy, protecting active ingredients from degradation, ensuring sustained antifungal activity. Nanoencapsulation not only reduces the required quantities of fungicides but also minimizes their toxicity and adverse effects on non-target organisms, while enhancing the direct efficacy of the antifungal compounds (Kumar et al., 2014; Huang et al., 2018). Furthermore, polymer nanospheres facilitate sustained release, offering advantages such as improved biosafety, selectivity, biodegradability, and economic viability, along with a reduced environmental footprint.

Among various materials, poly-ε-caprolactone (PCL), an aliphatic semi-crystalline polyester, is particularly notable for its excellent agricultural properties. PCL is biocompatible, exhibits low mammalian toxicity, undergoes rapid degradation, and can be engineered to produce pores of specific sizes, enabling the controlled release of encapsulated active compounds (Woodruff and Hutmacher, 2010). Furthermore, PCL demonstrates high encapsulation efficiency for plant essential oils, facilitating their sustained release in agricultural applications (Pinto et al., 2016). The combination of PCL with essential oils has shown significant potential in enhancing antifungal activity, making it a key rea of focus in pesticide research (Peres et al., 2020; Rosato et al., 2023). These findings underscore the potential of PCL-encapsulated essential oils for combating fungal pathogens in agricultural systems. However, the application of *Ss*EO as a fungal inhibitor in agricultural settings remains underexplored, highlighting the need for further investigation.

**SCHEME 1:**
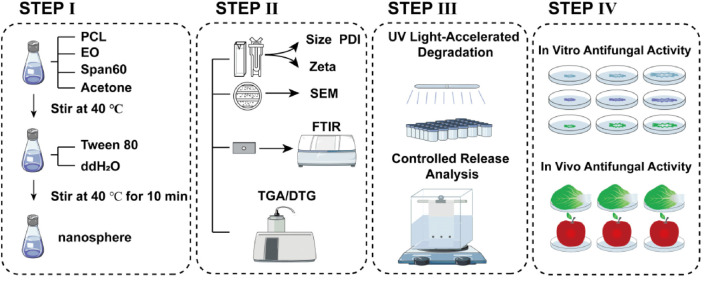
Schematic representation of the characterization and antifungal efficacy of PCL nanoencapsulated *Ss*EO.

This study explores a sustainable antifungal solution to combat environmental and agricultural issues stemming from chemical fungicide overuse. We investigate plant-derived nanotechnology, specifically *Ss*EO encapsulated in PCL nanospheres, as an eco-friendly crop protection alternative. We hypothesize that PCL nanoencapsulation will improve *Ss*EO’s stability, photoprotection, and antifungal efficacy due to the sustained release and biodegradability. We prepared and characterized SsEO-loaded PCL nanospheres (Scheme 1) and assessed their antifungal activity against five major plant pathogens, including *S. sclerotiorum* and *V. mali*, through *in vitro* and *in vivo* assays. The anticipated results will highlight the benefits of integrating natural bioactives with nanotechnology, promoting sustainable agriculture and providing a less environmentally harmful substitute for traditional fungicides.

## 2 Materials and methods

### 2.1 Materials

PCL (average molecular weight of 45,000) and potato dextrose agar (PDA) medium were procured from Macklin Biochemical Co., Ltd. and Sangon Biotech Co., Ltd., both located in Shanghai, China. Additionally, Span 60 (sorbitan 60 monostearate) and Tween 80 (polysorbate 80) were also obtained from Sangon Biotech Co., Ltd.

### 2.2 Chemical analysis of the *Ss*EO

In this study, the chemical composition of the *Ss*EO was analyzed to identify its specific compounds. Gas chromatograph (GC; TRACE 1300, United States) was employed for the analysis, with a carrier flow rate of 1 mL/min, and an inlet temperature set to 250°C. The temperature program for the GC analysis initiated at 50°C, increased to 100°C at a rate of 15°C /min, and further elevated to 200°C at a rate of 10°C /min.

Additionally, the content of *cis*-abienol in *Ss*EO was quantified using high-performance liquid chromatography (HPLC, Agilent 1260, Agilent, Santa Clara, United States) equipped with a UV-vis detector set at 237 nm. An Eclipse XDB-C18 column (4.6 mm × 250 mm, 5 μm film thickness, Agilent) was utilized, with a mobile phase consisting of methanol, ethanol and a 0.1% (v/v) aqueous acetic acid solution in a ratio of 18:71:11. The HPLC analysis was performed at a flow rate of 0.8 mL/min and a column temperature of 30°C.

### 2.3 *Ss*EO nanosphere preparation and characterization

Nanospheres were prepared using the preformed polymer nanoprecipitation method. The organic phase, comprising 150 mg of PCL biopolymer, 100 mg of Span 60, 10 mL of acetone (organic solvent) and varying quantities of essential oil, was prepared at 40°C and under continuous stirring. This organic phase was then gradually poured into an aqueous phase containing 100 mg of Tween 80 and 20 mL of distilled water, followed by stirring for 10 min to ensure stabilization. Subsequently, Water and organic solvents were removed using a rotary evaporator to obtain the final colloidal suspension.

Four formulations NS1, NS2, NS3, and NS4 were prepared (Table 1), containing 0, 50, 100, and 250 mg of essential oil, respectively. To characterize the NS1, NS2, NS3, and NS4 nanospheres, we evaluated their mean particle size, polydispersity index (PDI), and zeta-potential at 25°C using dynamic light scattering (DLS) with a Zeta Sizer Nano ZS90 (Malvern Instruments, Malvern, United Kingdom).

**TABLE 1:** Formulation of PCL nanospheres prepared with *Ss*EO.

Formulations	PCL (mg)	Span 60 (mg)	EO (mg)	Acetone (mL)	Tween 80 (mg)	Water (mL)
NS1	150	100	0	10	100	20
NS2	150	100	50	10	100	20
NS3	150	100	100	10	100	20
NS4	150	100	250	10	100	20

### 2.4 Encapsulation efficiency

The filtration–centrifugation method was employed to determine the quantity of the amount of nanoencapsulated *Ss*EO. Aliquots of the nanoparticle colloidal suspensions were transferred into tubes equipped with 0.22 μm pore cellulose acetate filters (Spin-X, Corning^®^ Inc., Corning, NY, United States) and centrifuged at 8,000 rpm and 20°C for 40 min. Subsequently, 250 μL of the ultrafiltrate was collected and analyzed by UV-vis spectroscopy. Encapsulation efficiency (EE%) was calculated using the following equation:


(1) $$E\,E\%=\frac{B-A}{B}\times 100\%$$

Where A (mg/mL) represents the amount of essential oil in the ultrafiltrate, and B (mg/mL) denotes the amount of essential oil in the suspension.

### 2.5 Nanoparticle morphology

The homogeneity of the colloidal suspension and the morphology of *Ss*EO nanospheres were evaluated at the Advanced Analysis and Testing Center of the Nanjing Forestry University.

The nanosphere suspension was frozen at −20°C for a duration of 24 h to ensure complete solidification. Subsequently, the frozen samples were transferred to a freeze dryer and subjected to sublimation for 48 h, yielding a dry, white powder. The dried samples were then mounted onto 12 mm diameter aluminum stubs and coated with a 2 nm layer of gold/palladium (Au/Pd) alloy using sputtering to enhance conductivity for analysis. The prepared samples were analyzed using Scanning electron microscopy (SEM) with a Quanta 200 instrument (FEI, Thermo Fisher Scientific, Waltham, MA, United States).

### 2.6 Fourier transform infrared spectroscopy and thermogravimetric analysis

Fourier transform infrared spectroscopy (FTIR) spectra was employed to evaluate the encapsulation of relative essential oils and to analyze the chemical structure of PCLs. FTIR spectra were acquired for essential oils, PCL, and the nanospheres using a Spectrum Two from PerkinElmer (Waltham, MA) equipped with the UATR Two accessory. Spectra were recorded at 4 cm^–1^ resolution.

Additionally, thermogravimetric analysis (TGA) and derivative thermogravimetric analysis (DTG) were conducted to investigate the thermal stability of the encapsulated essential oils and the composition of the nanosphere shells. The TGA measurements were performed using a Hitachi STA 7200 thermal analysis system (Ibaraki, Japan) under a controlled nitrogen atmosphere with a flow rate of 200 mL/min. The temperature was increased at a rate of 10°C /min over a range of 30–600°C.

### 2.7 UV light-accelerated degradation

The photostability of non-encapsulated and nanoencapsulated essential oils was evaluated using an ultraviolet chamber emitting light at a wavelength of 254 nm. Aliquots of 1 mL per sample were exposed to the UV chamber for 0, 1, 2, 3, 4, 5, 6, 7, 8, 9, and 10 h exposure intervals. Subsequently, the essential oil was extracted through vortexing and examined using by UV-vis spectrophotometry to evaluate the photostability of the essential oil.

### 2.8 Controlled release analysis of nanospheres

The release profile of the nanoparticles was determined using the inverse dialysis technique. 10 mL of nanoparticle suspension was mixed with 100 mL of 0.5% Tween 80 aqueous solution. 1 mL of this mixture was then transferred to a dialysis bag (MM 1200D cellulose membrane, Sigma Aldrich, Sintra, Portugal) and stirred continuously at room temperature. *Ss*EO release was quantified using a UV-vis photometer by sampling at fixed intervals (0, 0.5, 1, 2, 3, 6, 9, 12, 24, 48, and 72 h).

### 2.9 *In vitro* antifungal activity

The antifungal activity of the essential oil-loaded nanospheres was evaluated against five agriculturally significant plant pathogenic fungi: *Rhizoctonia solani, Gibberella zeae*, *Phytophthora infestans*, *Sclerotinia sclerotiorum*, and *Valsa mali*, using the mycelial growth rate inhibition assay. Essential oil nanospheres and blank control nanospheres were incorporated into potato dextrose agar (PDA) medium at various concentrations (100–750 mg/L), and the mixture was poured into sterile Petri dishes. Mature fungal cultures were punched at the colony margin and transferred to the prepared PDA plates. Plates were incubated at 28°C, and the colony diameters were measured after sufficient growth. The inhibition rate was calculated, and the half-maximal effective concentration (EC_50_) values were determined to assess antifungal efficacy.

The relative inhibition *I* (%) was calculated using the following equation:


(2) $$I\left(\%\right)=\frac{C-T}{C-5}\times 100\%$$

Where *I* is the inhibition rate, *C* is the colony diameter (mm) of the control group and *T* is the colony diameter (mm) of the test group.

### 2.10 *In vivo* antifungal activity

For the protective efficacy experiment, the nanospheres were uniformly sprayed onto apples in equal volumes, with an equivalent amount of 0.5% Tween 80 aqueous solution serving as a blank control. After 24 h, 5 mm agar blocks containing mycelium were inoculated onto the apples. The average diameter of the resulting lesions was measured 11 days post-inoculation. For curative efficacy experiments, 5 mm agar blocks with mycelium were first inoculated onto apples. After 24 h, the nanospheres were sprayed onto the apples, with an equal volume of 0.5% Tween 80 aqueous solution used as a blank control. The mean lesion diameter was measured on day 11 following inoculation.

The protective activity and curative activity (%) were calculated using the following equation:


(3) $$\mathrm{Protective}\mathrm{activity}/\mathrm{curative}\mathrm{activity}\left(\%\right)=\frac{c-t}{c-5}\times 100\%$$

Where *c* represents the colony diameter (mm) of the control group and *t* denotes the colony diameter (mm) of the test group.

### 2.11 Statistical analysis

The data presented in the figures are expressed as the mean ± standard deviation (SD). Statistical analysis of the experimental data was conducted using Origin 2021.

## 3 Results and discussion

### 3.1 Chemical analysis of the *Ss*EO and evaluation of antifungal components

The chemical analysis of the *Ss*EO was conducted using GC-MS (Supplementary Figure S1). The analysis revealed that *Ss*EO is predominantly composed of terpenes (Table 2), with monoterpenes constituting the dominant group. Notably, limonene accounts for 8.91% of the total composition, followed by α-myrcene at 5.57% and α-pinene at 3.26%. These monoterpenes are recognized for their aromatic and antioxidant properties, which likely contribute to the overall efficacy of *Ss*EO (Obermeier et al., 2025). In addition to terpenes, *Ss*EO contains alcohols such as linalool (11.48%) and cyclohexanol (7.29%), which are associated with antifungal and sedative effects, further enhancing the oil’s potential therapeutic properties (Tang et al., 2024). A significant presence of *cis*-abienol, a terpene compound with well-documented antifungal activity, was found at 3.8%, highlighting its potential role in crop disease management. The *Ss*EO also contains trace amounts of other compounds such as longicyclene, were also identified, which may provide additional health benefits. Overall, *Ss*EO exhibits a complex and diverse chemical profile, with key components suggesting antioxidant, antifungal, and sedative properties. This composition positions *Ss*EO as a promising, environmentally friendly alternative for managing crop diseases and reducing dependence on chemical fungicides.

**TABLE 2:** Chemical components of the *Ss*EO.

No.	Compounds	Fomula	MW	Relative percentage
1	α-Pinene	C_10_H_16_	136	3.26
2	Camphene	C_10_H_16_	136	0.15
3	α-Phellandrene	C_10_H_16_	136	0.23
4	α-Myrcene	C_10_H_16_	136	5.57
5	Carane	C_10_H_18_	138	1.94
6	Thujone	C_10_H_16_	136	2.05
7	Cyclofenchene	C_10_H_16_	136	0.57
8	D-Limonene	C_10_H_16_	136	8.91
9	Linalool	C_10_H_18_O	154	11.48
10	1-2-Dihydrolinalool	C_10_H_20_O	156	0.58
11	(+)-2-Bornanone	C_10_H_16_O	152	2.52
12	Menthone	C_10_H_18_O	154	3.13
13	Menthol	C_10_H_20_O	156	0.60
14	Cyclohexanol,	C_10_H_20_O	156	4.01
15	α-Terpineol	C_10_H_18_O	154	0.96
16	Cyclohexanone	C_10_H_16_O	152	0.17
17	Piperitone	C_10_H_16_O	152	0.13
18	Menthol acetate	C_12_H_22_O_2_	198	0.57
19	Safrole	C_10_H_10_O_2_	162	0.62
20	Thymol	C_10_H_14_O	150	6.51
21	α-Terpinyl acetate	C_12_H_20_O_2_	196	9.70
22	Longicyclene	C_15_H_24_	204	7.29
23	(+)-Sativen	C_15_H_24_	204	3.22
24	Longifolene	C_15_H_24_	204	5.02
25	Caryophyllene	C_15_H_24_	204	3.01
26	*cis*-α-Farnesene	C_15_H_24_	204	1.25
27	4,11,11-Trimethyl-8-methylenebicyclo [7.2.0] undec-3-ene	C_15_H_24_	204	0.50
28	Humulene	C_15_H_24_	204	2.89
29	2-epi-trans-β-caryophyllene	C_15_H_24_	204	0.78
30	(-)-α-Cedrene	C_15_H_24_	204	0.24
31	α-Himachalene	C_15_H_24_	204	0.91
32	Naphthalene	C_15_H_24_	204	0.82
33	Caryophyllene oxide	C_15_H_24_O	220	0.87
34	2-[4-methyl-6-(2,6,6-trimethylcyclohex-1-enyl) hexa-1,3,5-trienyl] cyclohex-1-en-1-carboxaldehyde	C_23_H_32_O	324	0.13
35	Caryophyllenyl alcohol	C_15_H_26_O	222	0.35
36	3,7-Cycloundecadien-1-ol, 1,5,5,8-tetramethyl-	C_15_H_26_O	222	0.17
37	Epiglobulol	C_15_H_26_O	222	0.30
38	caryophylla-4(12),8(13)-dien-5α-ol	C_15_H_24_O	220	0.23
39	Isoaromadendrene epoxide	C_15_H_24_O	220	0.24
40	Camphorene	C_20_H_32_	272	4.15
41	Isopropyl myristate	C_17_H_34_O_2_	270	0.15
42	*cis*-Abienol	C_20_H_34_O	290	3.80

### 3.2 Properties of the PCL-*Ss*EO nanospheres

**FIGURE 1:**
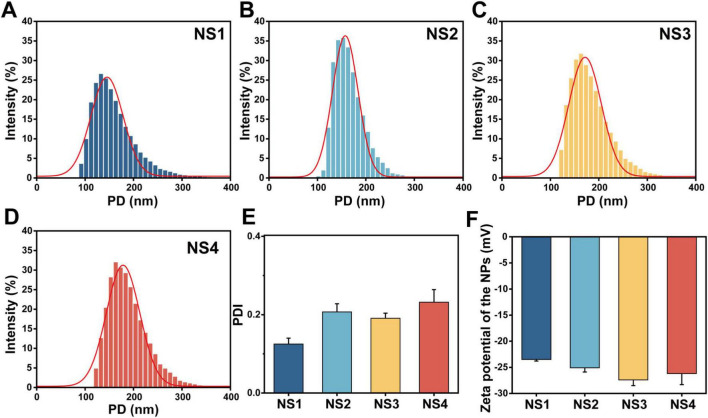
Characterization of nanoencapsulated *Ss*EO. **(A)** NS1 prepared without *Ss*EO. **(B)** NS2 prepared with 50 mg of *Ss*EO. **(C)** NS3 prepared with 100 mg of *Ss*EO. **(D)** NS4 prepared with 250 mg of *Ss*EO. **(E)** PDI of nanoencapsulated *Ss*EO. **(F)** Zeta potential of nanoencapsulated *Ss*EO. PD, particle diameter; PDI, polydispersity index.

In this study, the preformed polymer nanoprecipitation method was employed to encapsulate essential oils in PCL nanospheres. The molecular weight (Mw) of PCL plays a critical role in this formulation: high Mw can cause solubility issues and unstable emulsions, (Cesari et al., 2020) whereas using lower Mw PCL (45,000) enhanced stability, increased yield, and produced a more consistent particle size distribution. In contrast, the use of lower Mw PCL (45,000) in this study enhanced stability and increased yield, resulting in a more consistent particle size distribution. The analysis of the nanosphere formulations (NS1, NS2, NS3, NS4) provided key insights into their particle size distribution, polydispersity index (PDI), and zeta potential, which are essential parameters for evaluating nanoparticle stability and uniformity. As shown in Figures 1A–D, NS1 exhibited the smallest average particle size around 100 nm, while NS4 displayed a larger average size around 180 nm. This trend suggests that increasing the essential oil content leads to larger particles sizes, consistent with previous studies on PCL nanospheres encapsulating various drugs (Pereira et al., 2018).

Despite the increase in particle size with higher essential oil content, the PDI values remained consistent across all formulations (ranging from 0.2 to 0.3; Figure 1E), demonstrating uniform dispersion of the nanospheres. Additionally, the zeta potential values were negative (from −23.5 to −27.4 mV; Figure 1F), suggesting good colloidal stability and a reduced tendency for aggregation, which aligns with findings in similar systems (Ahlin et al., 2002). Overall, these results confirm that the preformed polymer nanoprecipitation method is effective for encapsulating essential oils in PCL nanospheres, yielding formulations with stable, uniform sizes and excellent colloidal stability. These properties lay the foundation for PCL-*Ss*EO nanospheres as a promising alternative to conventional fungicides and pharmaceutical formulations. (Pei et al., 2025).

### 3.3 Characterization of the PCL-*Ss*EO nanospheres

**FIGURE 2:**
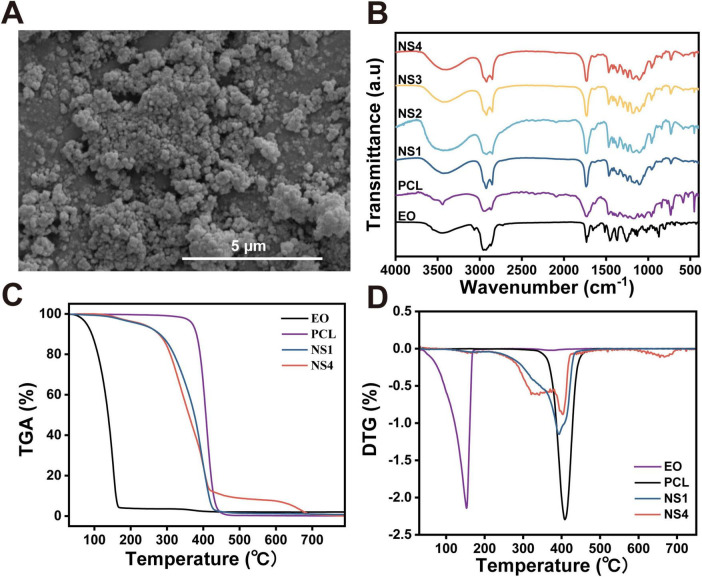
Different properties of nanoencapsulated *Ss*EO. **(A)** Scanning electron microscopic of nanospheres of *Ss*EO. **(B)** FTIR spectra of nanospheres, PCL, and *Ss*EO. TGA **(C)** and DTG **(D)** thermograms of NS1 (unloaded with *Ss*EO), NS4 (loaded with *Ss*EO), PCL and *Ss*EO. TGA, thermogravimetric analysis; DTG, derivative thermogravimetric analysis.

To further investigate the morphology of the nanospheres, SEM was employed to analyze their shape, surface texture, and diameter. The SEM images revealed that all formulations exhibited spherical nanospheres with uniform surfaces and shapes. Figure 2A showed that the PCL nanoencapsulated *Ss*EO nanospheres displayed smooth, crack-free surfaces, indicating the successful formation of a homogeneous polymer shell around the essential oil. These SEM findings confirm that the nanospheres possess the desired characteristics of stability, uniformity, and smooth encapsulation.

The FTIR analysis was conducted to identify the encapsulated essential oils and evaluate the shell composition of the nanospheres by analyzing their characteristic vibrational peaks. As shown in Figure 2B, the FTIR spectra of the prepared nanospheres, the PCL polymer, and the encapsulated essential oils were compared. A prominent peak at 1,720 cm^–1^, corresponding to the carbonyl C = O stretching vibration, was observed in the PCL spectrum, which is characteristic of polyesters such as PCL (Phillipson et al., 2014). Additionally, a symmetrical C-O-C stretching vibration peak at 1,170 cm^–1^ was detected, consistent with the presence of saturated esters (Nanni et al., 2019). These peaks confirmed the incorporation of the PCL structure within the nanospheres. The FTIR spectra of all nanosphere formulations (NS1, NS2, NS3, and NS4) exhibited the intense 1,720 cm^–1^ peak, indicative of the PCL carboxyl group stretching, further confirming the presence of PCL in the nanosphere shell (Elzein et al., 2004). These results demonstrate that the nanospheres retain the characteristic features of PCL, even after the incorporation of essential oils.

The essential oil, as summarized in Table 1, exhibited several distinct peaks in its FTIR spectrum. A peak at 1,732 cm^–1^, corresponding to the carbonyl (C = O) stretching vibration, is characteristic of aldehydes and ketones. Another peak at 1,655 cm^–1^ was attributed to C = C stretching, indicative of olefins. Additionally, several other bands associated with olefins and alcohols, including C-O stretching, C = C stretching, OH bending, and C-H stretching, were observed in the essential oil spectra. The presence of these peaks in the FTIR spectrum of the encapsulated nanospheres confirmed the successful incorporation of the essential oil into the PCL nanospheres. Furthermore, an intense peak at 2,870 cm^–1^, representing the stretching of methyl groups, was observed in both the essential oil and PCL spectra (Ayazi et al., 2025). A slight shoulder at lower frequencies, corresponding to characteristic peaks of the essential oil in this spectral region, further supported the encapsulation of the oil within the PCL shell. Additionally, a broad band ranging from 3,250 to 3,750 cm^–1^, typically associated with OH groups in the essential oil, was observed (Sahiner et al., 2018). This broad band provided further evidence of the encapsulation of essential oil components within the PCL nanospheres.

The TGA of the nanospheres and their components is presented in Figures 2C,D. PCL exhibited significant mass loss starting at approximately 390°C due to thermo-oxidative degradation. In contrast, *Ss*EO showed minimal mass loss between 50 and 100°C, with a rapid increase in mass loss around 11°C, likely resulting from the volatilization and degradation of its components (Pinto et al., 2016). The nanospheres, however, remained thermally stable up to 280°C, with no significant mass loss, as the PCL shell effectively insulated the essential oil. In Figure 2D, NS4, loaded with *Ss*EO, exhibited accelerated weight loss between 290 and 380°C compared to NS1, indicating the release and volatilization of the *Ss*EO from the nanospheres. These results demonstrate that the PCL nanosphere shell provided thermal stability, effectively encapsulating the essential oil until higher temperatures, at which point the oil is released.

### 3.4 Analysis of UV light accelerated degradation and *in vitro* release.

Essential oils are highly susceptible to environmental factors such as light, heat, and moisture, which can trigger degradation reactions that compromise their preservation and biological efficacy (Maia et al., 2019). These degradation reactions often alter the chemical structure of essential oils, diminishing their ability to inhibit fungal growth (Liu et al., 2014). In this study, the efficacy of poly-ε-caprolactone (PCL) nanospheres in protecting essential oils from photodegradation was evaluated.

**FIGURE 3:**
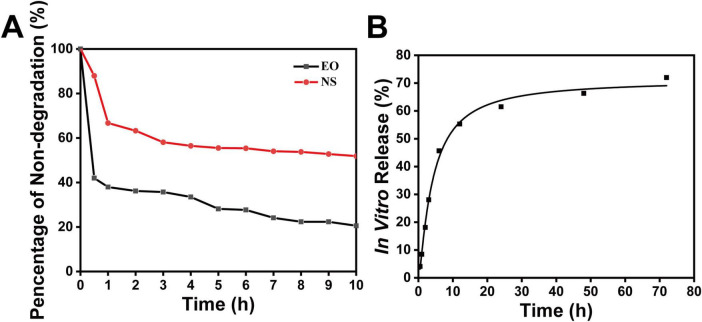
UV-Accelerated degradation and *in vitro* release of *Ss*EO nanospheres. **(A)** UV-accelerated degradation of free *Ss*EO and nanoencapsulated *Ss*EO (NS4). **(B)** *In vitro* release characteristics of *Ss*EO from nanospheres (NS) using the dialysis pocket diffusion technique. Each data point represents the average of three different NS batches.

As shown in Figure 3A, the degradation of free *Ss*EO and nanoencapsulated *Ss*EO (NS4) was compared under UV light exposure. The photodegradation experiment demonstrated a significant difference in UV stability between free *Ss*EO and nanoencapsulated *Ss*EO. After 0.5 h of UV exposure, free SsEO showed a rapid degradation rate of 58.0%, whereas nanoencapsulated *Ss*EO exhibited only 12.1% degradation under the same conditions. This substantial contrast underscores the protective effect of nanoencapsulation in reducing UV-induced degradation. With extended exposure to 1 h, degradation levels stabilized for both samples, with free *Ss*EO reaching a cumulative degradation rate of 63.1%, compared to 33.3% for the nanoencapsulated form. Free *Ss*EO exhibited 79.5% photodegradation after 10 h of exposure, while nanoencapsulated *Ss*EO showed only 48.2% degradation. This significant difference demonstrates that the PCL nanospheres effectively protect the essential oil from UV-induced oxidation and degradation. The encapsulation process preserved volatile components within the nanoparticles, thereby enhancing the photostability of *Ss*EO. These findings are consistent with prior research, which emphasize the critical role of nanoparticle encapsulation in preventing photodegradation of sensitive plant compounds (Christofoli et al., 2015; Pereira et al., 2018).

Further investigation into the *in vitro* release characteristics of *Ss*EO from the nanospheres (Figure 3B) revealed that the release mechanism follows a typical Fickian-type diffusion pattern, which is common for PCL-based delivery systems. Initially, 55.3% of *Ss*EO was rapidly released within 12 h, followed by a slower, sustained release over the subsequent 60 h. This release pattern indicates that the nanospheres provide both an initial burst release and prolonged release, which may enhance antifungal efficacy while minimizing side effects associated with rapid drug release (Dash and Konkimalla, 2012). The slow release after the first 12 h is likely attributed to the hydrolytic instability of the aliphatic ester bonds in PCL, which undergo gradual degradation in aqueous environments (Loureiro et al., 2020). This hydrolytic process leads to the slow breakdown of PCL, resulting in a reduction in the polymer’s molecular weight and eventual disintegration of the nanosphere shell. The decrease in molecular weight further destabilizes the shell, facilitating the release of the remaining essential oils. In conclusion, the combination of photodegradation protection and sustained release behavior positions the PCL-*Ss*EO nanospheres as a promising system for controlled drug delivery, offering prolonged antifungal activity with reduced side effects from rapid release. The protection against UV degradation and the controlled release dynamics highlight the potential of these nanospheres for various applications, including sustainable crop protection and therapeutic uses.

### 3.5 *In vitro* analysis of antifungal property

**FIGURE 4:**
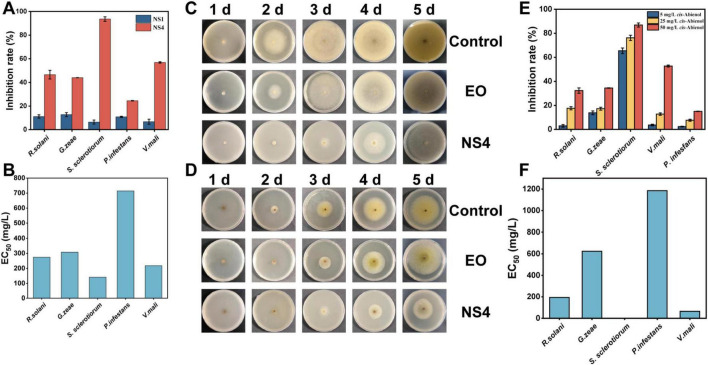
Analysis of *In vitro* antifungal efficacy of the nanospheres. **(A)** *In vitro* antifungal results of NS1 and NS4. **(B)** EC_50_ of nanospheres of *Ss*EO against five fungi. **(C)** Images of the PDA plates of antifungal activities to *S. sclerotiorum*. **(D)** Images of the PDA plates of antifungal activities to *V. mali*. **(E)** Analysis of antifungal efficacy of *cis*-abienol. **(F)** EC_50_ of nanospheres of *cis*-abienol against five fungi.

The antifungal efficacy of the nanospheres was evaluated by assessing their inhibitory effects on the mycelial growth of five plant pathogenic fungi (Yang et al., 2019). The preliminary bioactivity tests demonstrated that the nanospheres exhibited moderate inhibitory activity against *R. solani* and *G. zeae*, while showing limited inhibitory effects against *P. infesfans* compared to the blank control group. However, the nanospheres displayed significant inhibitory effects against *S. sclerotiorum* and *V. mali*, achieving relative inhibition rates of 56.9% for *V. mali* and 93.6% for *S. sclerotiorum*, as shown in Figure 4A. These results indicate that the nanospheres effectively inhibit the growth of specific fungal pathogens. The observed antifungal activity can be attributed to the terpenoids present in the essential oil fractions, which are abundant in the essential oils of the Asteraceae family (Zeng et al., 2021).Terpenes in essential oils are known to disrupt fungal cell membrane integrity, impairing membrane fluidity and thereby inhibiting fungal growth (Parker et al., 2022). While free essential oils initially demonstrated antifungal effects comparable to the nanospheres, their efficacy diminished after 3 days. In contrast, the nanospheres maintained significant antifungal activity over time, as shown in Figure 4C, highlighting the enhanced stability and sustained release of the nanoencapsulated essential oils compared to free essential oils. This sustained antifungal activity suggests that nanoencapsulation improves the long-term efficacy of *Ss*EO as a natural antifungal agent.

Based on these findings, *S. sclerotiorum* and *V. mali* were selected for further evaluation of the fungicidal activity of *Ss*EO nanospheres. The EC_50_ values for the *Ss*EO nanospheres were determined as 141.4 mg/L for *S. sclerotiorum* and 218.2 mg/L for *V. mali* (Figure 4B). These values highlighted the antifungal potency of the nanoencapsulated *Ss*EO, which was comparable to the effects observed with other plant extracts. In the antifungal tests against *V. mali*, the *Ss*EO nanospheres exhibited a high inhibition rate of 56.9%, compared to less than 40% for the commercial fungicide Ningnanmycin (Chen et al., 2022). Furthermore, the antifungal activity of *Ss*EO nanospheres was comparable to that of the positive control, 50 mg/L Boscalid, but their antifungal effect was superior. For *V. mali*, *Ss*EO nanospheres demonstrated 56.9% inhibition, surpassing Boscalid’s 35.8% inhibition, while for *S. sclerotiorum*, the 93.6% inhibition by *Ss*EO nanospheres also exceeded Boscalid’s 83.3% inhibition. This underscores the potential of *Ss*EO nanospheres as an effective alternative to traditional chemical fungicides. Additionally, the sustained antifungal effect of the nanospheres, indicative of their slow-release properties, is crucial for their practical applications (Ni et al., 2018). This sustained release was particularly evident in the mycelial inhibition tests on both *S. sclerotiorum* and *V. mali* (Figures 4C,D).

For *S. sclerotiorum*, the colonies on PDA plates exhibited typical growth patterns, transitioning from fluffy white mycelium to a dense mat with a cottony texture. As the colonies matured, distinct sclerotia (compact, black structures) appeared within the mycelium (Shang et al., 2024). In the groups treated with *Ss*EO and *Ss*EO nanospheres, fungal growth was significantly reduced, with sparse mycelial mass and hindered sclerotia formation, achieving a 93.6% mycelial inhibition rate. Similarly, for *V. mali*, the colonies initially appeared as white cottony patches that spread radially, eventually forming dense mycelial mats. As the colonies matured, pigmentation changed, and small yellow or green hyphae emerged. In the *Ss*EO and *Ss*EO nanosphere-treated groups, the growth was notably reduced, and fewer yellow or green hyphae were observed (Xu et al., 2025). While free *Ss*EO showed strong initial inhibition that weakened over time, the *Ss*EO nanospheres maintained a sustained inhibitory effect, with a relative inhibition rate of 56.9%. This consistent and prolonged activity further underscores the potential of *Ss*EO nanospheres as an effective and sustainable solution for plant pathogen management.

The antifungal efficacy of five terpenoids, major components of *Ss*EO, was evaluated at three different concentrations: 5, 25, and 50 mg/L. The results of the plate inhibition assays against five plant pathogenic fungi were presented in Figure 4E. Among the terpenoids tested, *cis*-abienol demonstrated significant antifungal activity. Compared to the blank control group, *cis*-abienol exhibited weaker inhibition against *R. solani*, *G. zeae* and *P. infesfans*, but showed substantial antifungal effects against *S. sclerotiorum* and *V. mali*. The relative inhibition rates were 44.44% against *V. mali* and 88.24% against *S. sclerotiorum*, indicating its strong inhibitory potential against these pathogens. The antifungal activity of *cis*-abienol was concentration-dependent within a certain range. The EC_50_ values of *cis*-abienol against *V. mali* and *S. sclerotiorum* were determined as 0.95 and 65.03 mg/L, respectively (Figure 4F). This suggested that *cis*-abienol exhibits high potency at lower concentrations against *V. mali* and a slightly lower, yet still significant, effect against *S. sclerotiorum*. In comparison, the antifungal effects of the other compounds were lower and poorly correlated with concentration (Supplementary Figure S2). Thus, *cis*-abienol is identified as the primary antifungal component in *Ss*EO, with promising potential for development into an efficient plant fungicide (Li et al., 2019; Zhang et al., 2022). This highlighted its significant role in the antifungal activity of *Ss*EO and suggests that it could be a key target for further research and application in plant disease management.

### 3.6 *In vivo* analysis of antifungal property

Based on the *in vitro* bioassay data, *V. mali* and *S. sclerotiorum* were selected for further investigation of the *in vivo* antifungal activity of nanospheres against plant pathogenic fungi. *V. mali*, which caused apple tree canker, was a serious disease that severely affected apples (Han et al., 2024). Therefore, apples were chosen for the *in vivo* inhibition experiments, with a 0.5% Tween 80 aqueous solution used as a negative control.

**FIGURE 5:**
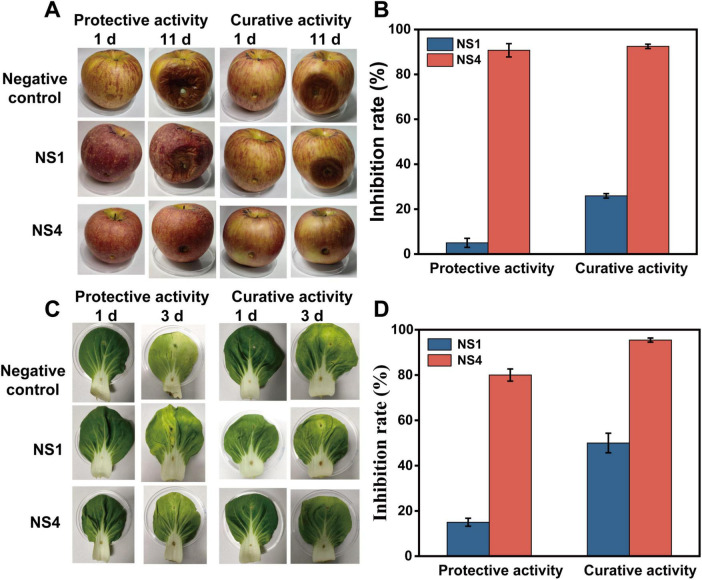
Analysis of *In vivo* antifungal efficacy of the nanospheres against *V. mali* on apples and pakchoi cabbage. **(A)** Images of *in vivo* antifungal results of NS1 and NS4 against *V. mali* on apples. **(B)** *In vivo* antifungal results of NS1 and NS4 against *V. mali*. **(C)** Images of *in vivo* antifungal results of NS1 and NS4 against *S. sclerotiorum* on pakchoi cabbage. **(D)** *In vivo* antifungal results of NS1 and NS4 against *S. sclerotiorum.*

As shown in Figure 5A, nanosphere NS1, which is unloaded with *Ss*EO, exhibited minimal inhibitory effects compared to the control. In contrast, apples treated with nanosphere NS4 (nanoencapsulated *Ss*EO) showed small disease patches. The relative inhibition rate of *V. mali* by nanosphere NS4 exceeded 90% in both protective and curative groups, while NS1, which did not contain *Ss*EO, displayed only a 22% inhibition rate in curative group (Figure 5B). In the curative group, the inhibition rate of *V. mali* by NS4 reached 92.5%, indicating that the PCL nanoencapsulated *Ss*EO has a significant inhibitory effect on *V. mali in vivo*. Interestingly, the timing of medicine administration (protective vs. curative groups) did not significantly affect the inhibitory effect. This result is consistent with the findings from the *in vitro* antifungal inhibition tests, where early administration demonstrated notable protective activity on apples (Wang et al., 2021). Moreover, while the disease spots in the control group spread across the apple surface, the spots on apples treated with NS4 showed no signs of spreading, further confirming the sustained antifungal effect and slow-release capability of the nanospheres. In summary, these results highlight the potent *in vivo* antifungal activity of the PCL-*Ss*EO nanospheres, with effective inhibition of *V. mali* in apples, demonstrating both protective and curative capabilities. The preparation of PCL nanospheres provides a sustained release mechanism during prevention or treatment, protecting the bioaccessibility of *Ss*EO and enhancing its therapeutic potential (Zhu et al., 2025).

*S. sclerotiorum* is a major plant pathogen that affects a wide range of crops and vegetables globally, infecting both monocotyledonous and dicotyledonous plants. (Desouky et al., 2025) In this study, pakchoi cabbage was selected for *in vivo* inhibition experiments with *S. sclerotiorum*, using a 0.5% Tween 80 aqueous solution as the negative control. As shown in the Figure 5C, the inhibition experiment on *S. sclerotiorum* demonstrated a significant effect on pakchoi cabbage leaves. Notably, the NS1 group (unloaded with *Ss*EO) showed no observable phytotoxicity compared to the negative control, confirming the safety of the PCL nanocarrier material for plant applications. In the NS4 group, there was noticeable reduction in *S. sclerotiorum* growth, with a clear sterile area around the inoculated sites on the leaves, which remained healthy and green. This was in stark contrast to the heavily infected control group, where yellow patches and white hyphae were visible, indicating severe fungal colonization. This observation suggests that nanosphere NS4, containing nanoencapsulated *Ss*EO, exhibits potent antifungal properties against *S. sclerotiorum* on the surface of pakchoi cabbage. In Figure 5D, nanosphere NS1 (unloaded with *Ss*EO) showed weaker antifungal effects compared to the control group. However, pakchoi cabbage leaves treated with NS4 showed fewer fungal spots, and the inhibition rate of *S. sclerotiorum* by NS4 was similar in both the protective and curative groups, with relative inhibition rate of 80.00 and 95.45%, respectively. This indicates that NS4 had a strong *in vivo* inhibitory effect on *S. sclerotiorum*, providing effective protection against the pathogen and confirming its potential as a natural antifungal agent for crop protection. Overall, the results highlight the promising efficacy of NS4 in managing *S. sclerotiorum* infections on pakchoi cabbage, demonstrating both protective and curative effects in real-world agricultural settings.

## 4 Conclusion

The *in vivo* antifungal activity of PCL nanospheres encapsulating *Ss*EO demonstrated potent inhibition against major plant pathogens, including *V. mali* and *S. sclerotiorum*. Nanoencapsulation significantly enhanced antifungal efficacy by protecting the essential oil from photodegradation and ensuring a sustained release profile, thereby prolonging its antifungal activity. PCL-*Ss*EO nanospheres significantly outperformed free essential oils and control treatments in both protective and curative applications, achieving over 90% inhibition of *S. sclerotiorum* and *V. mali* on the surfaces of apple and pakchoi cabbage. The slow release and protective properties of these nanospheres position them as an environmentally friendly and sustainable alternative to conventional chemical fungicides. These findings underscore the potential of PCL-*Ss*EO nanospheres for effective agricultural pest and disease management, offering a promising solution for enhanced crop protection with minimal environmental impact.

## Data Availability

The original contributions presented in the study are included in the article/Supplementary material, further inquiries can be directed to the corresponding author.

## References

[B1] Ahlin P. Kristl J. Kristl A. Vrečer F. (2002). Investigation of polymeric nanoparticles as carriers of enalaprilat for oral administration. *Int. J. Pharm.* 239 113–120. 10.1016/S0378-5173(02)00076-5 12052696

[B2] Ayazi D. Zandi M. Ganjloo A. Dardmeh N. (2025). Bilayer bioactive film based on sage seed gum-pea protein isolate/electrospun zein loaded with cumin essential oil: Characterization, biodegradability and release study. *J. Food Meas. Charact.* 19 1489–1496. 10.1007/s11694-024-03092-7

[B3] Cesari A. Loureiro M. V. Vale M. Yslas E. I. Dardanelli M. Marques A. C. (2020). Polycaprolactone microcapsules containing citric acid and naringin for plant growth and sustainable agriculture: Physico-chemical properties and release behavior. *Sci. Total Environ.* 703:135548. 10.1016/j.scitotenv.2019.135548 31767319

[B4] Chen Y.-Z. Wang S.-R. Li T. Zhang G.-C. Yang J. (2022). Antifungal Activity of 6-mthylcoumarin against valsa mali and its possible mechanism of action. *JoF* 9:5. 10.3390/jof9010005 36675826 PMC9861068

[B5] Cheung N. Tian L. Liu X. Li X. (2020). The destructive fungal pathogen botrytis cinerea—Insights from genes studied with mutant analysis. *Pathogens* 9:923. 10.3390/pathogens9110923 33171745 PMC7695001

[B6] Christofoli M. Costa E. C. C. Bicalho K. U. de Cássia Domingues V. Peixoto M. F. Alves C. C. F. (2015). Insecticidal effect of nanoencapsulated essential oils from *Zanthoxylum rhoifolium* (Rutaceae) in *Bemisia tabaci* populations. *Ind. Crop Prod.* 70 301–308. 10.1016/j.indcrop.2015.03.025

[B7] Dash T. K. Konkimalla V. B. (2012). Poly-ε-caprolactone based formulations for drug delivery and tissue engineering: A review. *J. Control Release* 158 15–33. 10.1016/j.jconrel.2011.09.064 21963774

[B8] Desouky M. M. Abou-Saleh R. H. Moussa T. A. A. Fahmy H. M. (2025). Nano-chitosan-coated, green-synthesized selenium nanoparticles as a novel antifungal agent against Sclerotinia sclerotiorum: in vitro study. *Sci. Rep.* 15:1004. 10.1038/s41598-024-79574-x 39762311 PMC11704303

[B9] Elzein T. Nasser-Eddine M. Delaite C. Bistac S. Dumas P. (2004). FTIR study of polycaprolactone chain organization at interfaces. *J. Colloid. Interf. Sci.* 273 381–387. 10.1016/j.jcis.2004.02.001 15082371

[B10] Fisher M. C. Henk D. A. Briggs C. J. Brownstein J. S. Madoff L. C. McCraw S. L. (2012). Emerging fungal threats to animal, plant and ecosystem health. *Nature* 484 186–194. 10.1038/nature10947 22498624 PMC3821985

[B11] Han P. Zhang R. Li R. Li F. Nie J. Xu M. (2024). MdVQ12 confers resistance to *Valsa mali* by regulating MdHDA19 expression in apple. *Mol. Plant Pathol.* 25:e13411. 10.1111/mpp.13411 38071459 PMC10788466

[B12] Huang B. Chen F. Shen Y. Qian K. Wang Y. Sun C. (2018). Advances in targeted pesticides with environmentally responsive controlled release by nanotechnology. *Nanomaterials* 8:102. 10.3390/nano8020102 29439498 PMC5853733

[B13] Kumar S. Bhanjana G. Sharma A. Sidhu M. C. Dilbaghi N. (2014). Synthesis, characterization and on field evaluation of pesticide loaded sodium alginate nanoparticles. *Carbohyd. Polym.* 101 1061–1067. 10.1016/j.carbpol.2013.10.025 24299874

[B14] Lawson S. K. Sharp L. G. Powers C. N. McFeeters R. L. Satyal P. Setzer W. N. (2020). Volatile compositions and antifungal activities of native American medicinal plants: Focus on the Asteraceae. *Plants* 9:126. 10.3390/plants9010126 31963839 PMC7020142

[B15] Li L. Wang X. Li X. Shi H. Wang F. Zhang Y. (2019). Combinatorial engineering of mevalonate pathway and diterpenoid synthases in *Escherichia coli* for cis -abienol production. *J. Agric. Food Chem.* 67 6523–6531. 10.1021/acs.jafc.9b02156 31117507

[B16] Li Y. Erhunmwunsee F. Liu M. Yang K. Zheng W. Tian J. (2022). Antimicrobial mechanisms of spice essential oils and application in food industry. *Food Chem.* 382:132312. 10.1016/j.foodchem.2022.132312 35158267

[B17] Liu P. Y. Li B. Liu H. D. Tian L. (2014). Photochemical behavior of fenpropathrin and λ-cyhalothrin in solution. *Environ. Sci. Pollut.* 21 1993–2001. 10.1007/s11356-013-2119-6 24019141

[B18] Loureiro M. V. Vale M. Galhano R. Matos S. Bordado J. C. Pinho I. (2020). Microencapsulation of Isocyanate in Biodegradable Poly(ε-caprolactone) Capsules and Application in Monocomponent Green Adhesives. *ACS Appl. Polym. Mater.* 2 4425–4438. 10.1021/acsapm.0c00535

[B19] Maia J. D. La Corte R. Martinez J. Ubbink J. Prata A. S. (2019). Improved activity of thyme essential oil (Thymus vulgaris) against Aedes aegypti larvae using a biodegradable controlled release system. *Ind. Crop. Prod.* 136 110–120. 10.1016/j.indcrop.2019.03.040

[B20] Meng F. Lei Y. Zhang Q. Li Y. Chen W. Liu D. (2022). Encapsulation of Zanthoxylum bungeanum essential oil to enhance flavor stability and inhibit lipid oxidation of C hinese- style sausage. *J. Sci. Food Agric.* 102 4035–4045. 10.1002/jsfa.11752 34997590

[B21] Miyazawa M. Tamura N. (2008). Characteristic odor components in the essential oil from yacón tubers (Polymnia sonchifolia Poepp. et Endl.). *J. Essent. Oil Res.* 20 12–14. 10.1080/10412905.2008.9699409

[B22] Nanni G. Heredia-Guerrero J. A. Paul U. C. Dante S. Caputo G. Canale C. (2019). Poly(furfuryl alcohol)-Polycaprolactone Blends. *Polymers* 11:1069. 10.3390/polym11061069 31226802 PMC6630956

[B23] Ni L. Rong S. Gu G. Hu L. Wang P. Li D. (2018). Inhibitory effect and mechanism of linoleic acid sustained-release microspheres on Microcystis aeruginosa at different growth phases. *Chemosphere* 212 654–661. 10.1016/j.chemosphere.2018.08.045 30173112

[B24] Obermeier F. Mutschlechner M. Haller S. Schöbel H. Strube O. I. (2025). Sustainable antimicrobial and antibiofilm strategies: Monoterpene-based compounds as potential substitute for low-molecular-weight biocides in the coating industry. *Eur. Polym. J.* 225 113734–113734.

[B25] Odukkathil G. Vasudevan N. (2013). Toxicity and bioremediation of pesticides in agricultural soil. *Rev. Environ. Sci. Biotechnol.* 12 421–444. 10.1007/s11157-013-9320-4

[B26] Parker R. A. Gabriel K. T. Graham K. D. Butts B. K. Cornelison C. T. (2022). Antifungal activity of select essential oils against *Candida auris* and their interactions with antifungal drugs. *Pathogens* 11:821. 10.3390/pathogens11080821 35894044 PMC9331469

[B27] Pei H. Wang M. Yang T. Li J. Sun S. Wang T. (2025). A hybrid of bimodal mesoporous silica and metal organic framework for intelligent co-delivery of dual-pesticide for synergistic controlling fungal disease and insect pest. *Colloid. Surf. A* 709 136140–136140. 10.1016/j.colsurfa.2025.136140

[B28] Pereira K. D. C. Quintela E. D. Da Silva D. J. Do Nascimento V. A. Da Rocha D. V. M. Silva J. F. A. (2018). Characterization of nanospheres containing *Zanthoxylum riedelianum* fruit esential oil and their insecticidal and deterrent activities against *Bemisia tabaci* (*Hemiptera: Aleyrodidae*). *Molecules* 23:2052. 10.3390/molecules23082052 30115840 PMC6222527

[B29] Peres M. C. De Souza Costa G. C. Dos Reis L. E. L. Da Silva L. D. Peixoto M. F. Alves C. C. F. (2020). In natura and nanoencapsulated essential oils from xylopia aromatica reduce oviposition of *Bemisia tabaci* in *Phaseolus vulgaris*. *J. Pest. Sci.* 93 807–821. 10.1007/s10340-019-01186-6

[B30] Phillipson K. Hay J. N. Jenkins M. J. (2014). Thermal analysis FTIR spectroscopy of poly(ε-caprolactone). *Thermochim. Acta* 595 74–82. 10.1016/j.tca.2014.08.027

[B31] Pinto N. D. O. F. Rodrigues T. H. S. Pereira R. D. C. A. Silva L. M. A. E. Cáceres C. A. Azeredo H. M. C. D. (2016). Production and physico-chemical characterization of nanocapsules of the essential oil from Lippia sidoides Cham. *Ind. Crop. Prod.* 86 279–288. 10.1016/j.indcrop.2016.04.013

[B32] Prem P. Naveenkumar S. Kamaraj C. Ragavendran C. Priyadharsan A. Manimaran K. (2024). Valeriana jatamansi root extract a potent source for biosynthesis of silver nanoparticles and their biomedical applications, and photocatalytic decomposition. *Green. Chem. Lett. Rev.* 17:2305142. 10.1080/17518253.2024.2305142

[B33] Rosato R. Napoli E. Granata G. Di Vito M. Garzoli S. Geraci C. (2023). Study of the chemical profile and anti-fungal activity against *Candida auris* of cinnamomum cassia essential oil and of its nano-formulations based on polycaprolactone. *Plants* 12:358. 10.3390/plants12020358 36679069 PMC9860731

[B34] Sahiner M. Sahiner N. Sagbas S. Fullerton M. L. Blake D. A. (2018). Fabrication of biodegradable poly (naringin) particles with antioxidant activity and low toxicity. *ACS Omega* 3 17359–17367. 10.1021/acsomega.8b02292

[B35] Shang Q. Jiang D. Xie J. Cheng J. Xiao X. (2024). The schizotrophic lifestyle of *Sclerotinia sclerotiorum*. *Mol. Plant. Pathol.* 25:e13423. 10.1111/mpp.13423 38407560 PMC10895550

[B36] Tang T. Zhong W. Tang P. Dai R. Guo J. (2024). Linalool’s multifaceted antimicrobial potential: Unveiling its 2 antimicrobial efficacy and immunomodulatory role against. *Drug Res.* 74 255–268. 10.1055/a-2321-9571 38968949

[B37] Trindade F. G. Taveira G. B. De Carvalho L. P. Moreira V. F. Feitoza R. B. B. Gomes V. M. (2023). Antifungal and antiparasitic activity of *Wunderlichia azulensis* (Asteraceae) roots. *Plant. Biosyst.* 157 658–669. 10.1080/11263504.2023.2186508

[B38] Van De Vel E. Sampers I. Raes K. (2019). A review on influencing factors on the minimum inhibitory concentration of essential oils. *Cri. Rev. Food Sci.* 59 357–378. 10.1080/10408398.2017.1371112 28853911

[B39] Wang W. Wang J. Wu F. Zhou H. Xu D. Xu G. (2021). Synthesis and biological activity of novel pyrazol-5-yl-benzamide derivatives as potential succinate dehydrogenase inhibitors. *J. Agric. Food Chem.* 69 5746–5754. 10.1021/acs.jafc.0c08094 33988994

[B40] Woodruff M. A. Hutmacher D. W. (2010). The return of a forgotten polymer—Polycaprolactone in the 21st century. *Prog. Polym. Sci.* 35 1217–1256. 10.1016/j.progpolymsci.2010.04.002

[B41] Wu C.-C. Huang S.-L. Ko C.-H. Chang H.-T. (2022). Antifungal sesquiterpenoids from *Michelia formosana* leaf essential oil against wood-rotting fungi. *Molecules* 27:2136. 10.3390/molecules27072136 35408536 PMC9000555

[B42] Xu Z. Wu B. Wu C. Chen Q. Niu Y. Shi Z. (2025). Acrylpimaric acid-modified chitosan derivatives as potential antifungal agents against Valsa Mali. *Carbohydr. Polym.* 52:123244. 10.1016/j.carbpol.2025.123244 39843115

[B43] Yang D. Zhao B. Fan Z. Yu B. Zhang N. Li Z. (2019). Synthesis and biological activity of novel succinate dehydrogenase inhibitor derivatives as potent fungicide candidates. *J. Agric. Food Chem.* 67 13185–13194. 10.1021/acs.jafc.9b05751 31697490

[B44] Zeng Z. Y. Li Q. Q. Huo Y. Y. Chen C. J. Duan S. S. Xu F. R. (2021). Inhibitory effects of essential oils from asteraceae plant against pathogenic fungi of panax notoginseng. *J. Appl. Microbiol.* 130 592–603. 10.1111/jam.14606 32026569

[B45] Zhang X. Zhu K. Shi H. Wang X. Zhang Y. Wang F. (2022). Engineering *Escherichia coli* for effective and economic production of cis-abienol by optimizing isopentenol utilization pathway. *J. Cleaner Prod.* 51:131310. 10.1016/j.jclepro.2022.131310

[B46] Zhu M. Chen D. Li C. Ouyang G. (2025). Discovery of berberine derivatives@chitosan nanoparticles for controlling plant canker diseases. *Food Hydrocolloids* 163:111054. 10.1016/j.foodhyd.2025.111054

