# Supplementary material for: Antifungal and biodegradable nanoencapsulation of Smallanthus Sonchifolius essential oil for improved stability and sustained release
Source: Front Microbiol. 2025 Jul 1;16:1626646. doi: 10.3389/fmicb.2025.1626646 (PMC12261442; doi:10.3389/fmicb.2025.1626646)
Supplement: Supplementary file 1 [file Supplementary_file_1.docx]

Antifungal and biodegradable nanoencapsulation of *Smallanthus Sonchifolius* essential oil for improved stability and sustained release

Jiajie Wang ^a^, Fei Wang^a^, Yu Zhang^a^, Xun Li^a*^

^a^ Co-Innovation Center for Efficient Processing and Utilization of Forest Resources, National Key Laboratory for the Development and Utilization of Forest Food Resources, College of Chemical Engineering, Nanjing Forestry University, Nanjing 210037, China.

Corresponding author at: College of Chemical Engineering, Nanjing Forestry University, No. 159 Longpan Road, Nanjing 210037, PR China.

E-mail: [xunli@njfu.edu.cn](mailto:xunli@njfu.edu.cn)

Tel: (+86)-025-85427396

Fax: (+86)-025-85428873


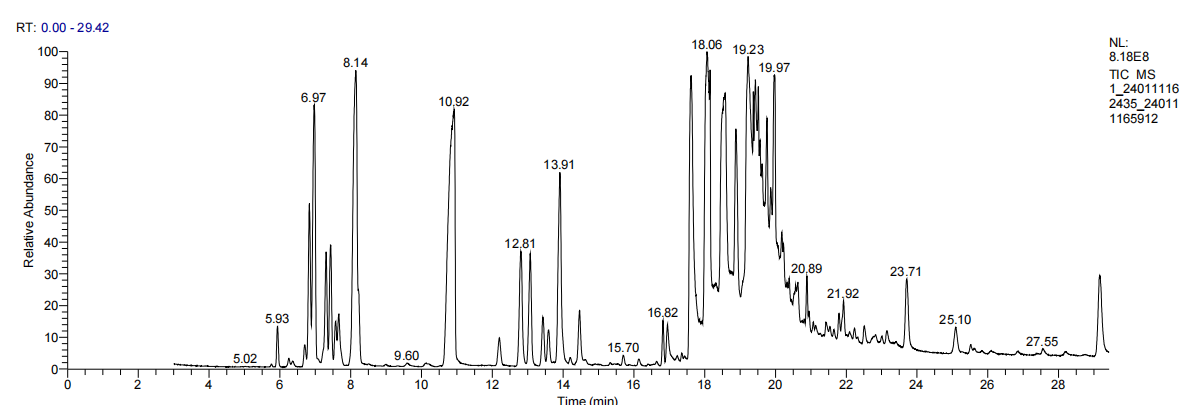


Figure S1 Gas Chromatography-Mass Spectrometry Analysis of the *Ss*EO


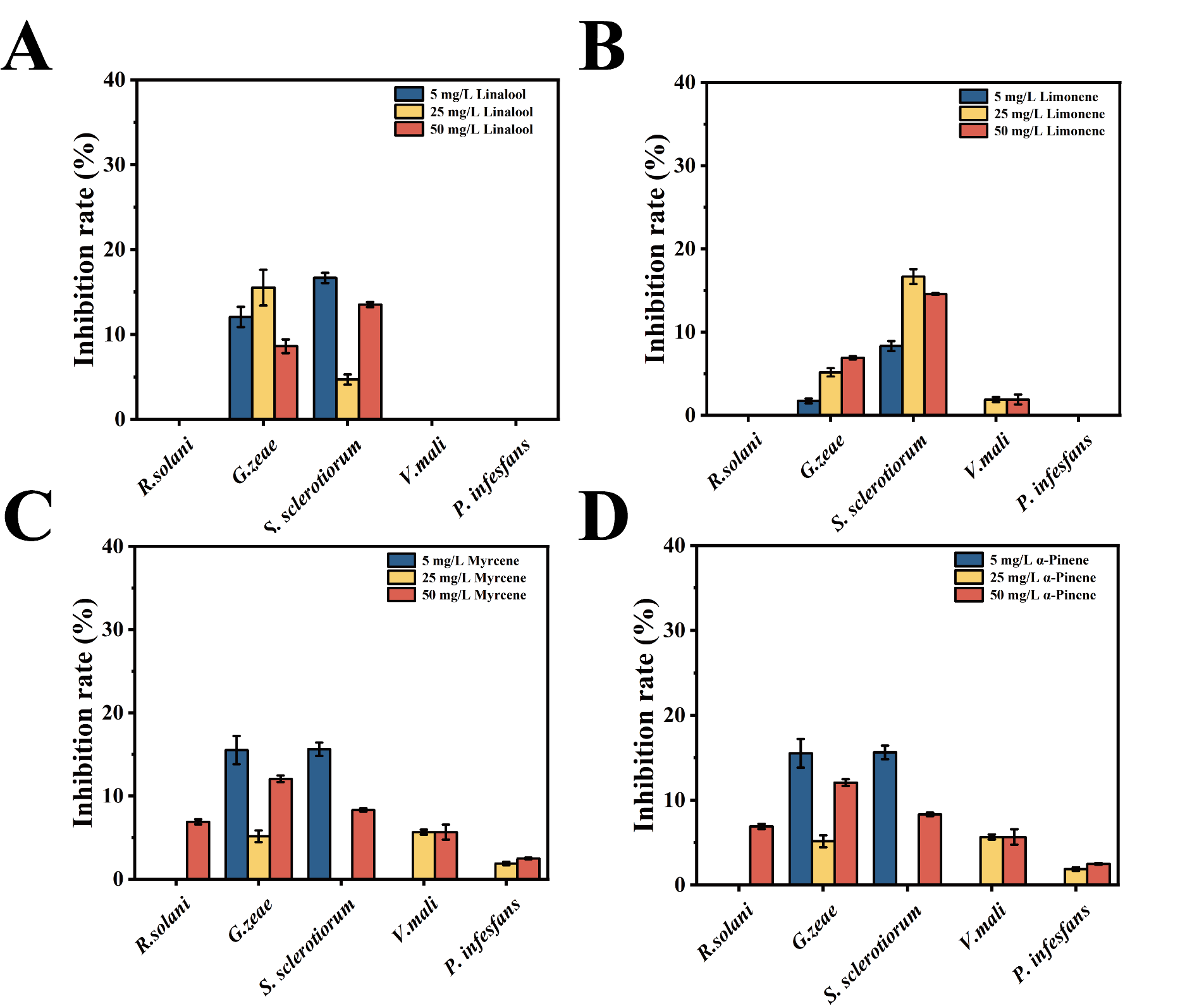


Figure S2 Analysis of antifungal efficacy of linalool(A), limonene(B), myrcene(C), α-Pinene(D).
